# Requiring smartphone ownership for mHealth interventions: who could be left out?

**DOI:** 10.1186/s12889-019-7892-9

**Published:** 2020-01-20

**Authors:** Krishna K. Bommakanti, Laramie L. Smith, Lin Liu, Diana Do, Jazmine Cuevas-Mota, Kelly Collins, Fatima Munoz, Timothy C. Rodwell, Richard S. Garfein

**Affiliations:** 1 Department of Medicine, University of California San Diego, 9500 Gilman Drive, La Jolla, CA USA; 2 Center for Gender Equity and Health, University of California San Diego School of Medicine, 9500 Gilman Drive, La Jolla, CA USA; 3 San Diego State University, 5500 Campanile Drive, San Diego, California 92182 USA; 4 Department of Family Medicine & Public Health, 9500 Gilman Drive, La Jolla, CA 92093 USA

**Keywords:** VDOT, DOT, Tuberculosis, mHealth, Smartphone

## Abstract

**Background:**

Mobile health (mHealth) interventions have the potential to improve health through patient education and provider engagement while increasing efficiency and lowering costs. This raises the question of whether disparities in access to mobile technology could accentuate disparities in mHealth mediated care. This study addresses whether programs planning to implement mHealth interventions risk creating or perpetuating health disparities based on inequalities in smartphone ownership.

**Methods:**

Video Directly Observed Therapy (VDOT) is an mHealth intervention for monitoring tuberculosis (TB) treatment adherence through videos sent by patients to their healthcare provider using smartphones. We conducted secondary analyses of data from a single-arm trial of VDOT for TB treatment monitoring by San Diego, San Francisco, and New York City health departments. Baseline and follow-up treatment interviews were used to assess participant smartphone ownership, sociodemographics and TB treatment perceptions. Univariate and multivariable logistic regression analyses were used to identify correlates of smartphone ownership.

**Results:**

Of the 151 participants enrolled, mean age was 41 years (range: 18–87 years) and 41.1% were female. Participants mostly identified as Asian (45.0%) or Hispanic/Latino (29.8%); 57.8% had at most a high school education. At baseline, 30.4% did not own a smartphone, which was similar across sites. Older participants (adjusted odds ratio [AOR] = 1.09 per year, 95% confidence interval [CI]: 1.05–1.12), males (AOR = 2.86, 95% CI: 1.04–7.86), participants having at most a high school education (AOR = 4.48, 95% CI: 1.57–12.80), and those with an annual income below $10,000 (AOR = 3.06, 95% CI: 1.19, 7.89) had higher odds of not owning a smartphone.

**Conclusions:**

Approximately one-third of TB patients in three large United States of America (USA) cities lacked smartphones prior to the study. Patients who were older, male, less educated, or had lower annual income were less likely to own smartphones and could be denied access to mHealth interventions if personal smartphone ownership is required.

## Background

Nonadherence to complicated medication regimens is a major cause of poor patient outcomes globally [1]. This is particularly evident in the treatment of infectious diseases, such as tuberculosis (TB). As the leading cause of death due to infectious diseases worldwide, TB affects nearly 2 billion people with 8.8 million new cases diagnosed each year [2, 3]. This has prompted the World Health Organization (WHO) and other agencies to prioritize the timely and effective treatment of TB via directly observed therapy (DOT) [2]. DOT is a component of case management in which patients travel to health clinics (or vice versa) and are observed by a healthcare worker ingesting each medication dose to ensure adherence and achieve treatment completion. Though DOT improves adherence, the effort required by patients and costs of DOT have proven to be a substantial barrier to broad implementation [4, 5]. Video DOT (VDOT) was developed to address these barriers while increasing patient autonomy throughout the treatment process and lowering treatment monitoring costs while also increasing patient adherence relative to DOT [6, 7]. In VDOT, patients video-record themselves taking their medications and securely transfer the videos to health workers for review. VDOT was initially piloted in the USA in 2010, where it was shown to be feasible in both high and low resource settings [3]. More recently, VDOT was found to be an acceptable alternative to DOT in Bangalore, South India, and Vietnam [8, 9].

Since TB disproportionately affects low-income populations, we assessed smartphone ownership among patients with TB who participated in an mHealth intervention to gain insights into other underserved populations.

Although existing literature provides support for feasibility and effectiveness of mHealth interventions for the treatment of TB and other diseases, some questions remain about its sustainability in resource-limited settings [5, 10]. For example, a recent study of daily smokers in Washington, D.C. who were enrolled in a mHealth-based smoking cessation intervention showed that mobile phone ownership was not a significant barrier to study participation. The authors found that none of the demographics they used to screen patients were significantly associated with smartphone ownership. However, participants were recruited by an online screener, which could itself exclude individuals who do not own a smartphone [11]. Studies that include participants based on their disease status and assess smartphone ownership are needed to determine which patients could be excluded from mHealth interventions if they are required to use their own smartphone.

It is important to understand whether mHealth interventions modeled after VDOT could potentially create new health disparities by disproportionately excluding patient groups that are less likely to own a smartphone. Since most studies evaluating smartphone-based interventions provide devices to the participants, it is important to understand which patients could be excluded if personal smartphone ownership is required. Given the growing interconnection between healthcare and technology, answering the question of who is more or less likely to own a smartphone will help inform the design of future, adequately resourced, sustainable mHealth applications and interventions.

## Methods

This analysis used baseline and follow-up data from a single-arm trial evaluating a VDOT smartphone application. The VDOT application was used to monitor TB treatment adherence among patients receiving care through health departments in three large USA cities with TB incidence rates (6.6–11.5 per 100,000 persons) that exceeded the national average (2.8 per 100,000 persons) [12]. The parent study was designed to assess the feasibility, acceptability and potential efficacy of the VDOT application for monitoring TB treatment adherence. Methods for the parent study were similar to those used in a previously published VDOT study that has been described elsewhere and described briefly below [3]. To avoid heterogeneity in the way the application performed on different mobile devices, all study participants were loaned a smartphone with service for the duration of the study regardless of whether or not they personally owned a smartphone; thus, smartphone ownership was not a study inclusion criterion. Written informed consent was obtained from all participants and the study protocol was approved by the Institutional Review Boards of the University of California San Diego, University of California San Francisco, the New York City Department of Health and Mental Hygiene, and the San Diego County Health and Human Services Agency Research Committee.

### Study Population & Eligibility

Participants were recruited through TB Control Programs in three major metropolitan health districts - New York City, San Francisco, and San Diego. TB Control Program staff at each site recruited individuals currently receiving treatment for confirmed or suspected TB. Eligibility criteria included: 1) ability to speak English or Spanish, 2) age at least 18 years, and 3) willing and able to provide informed consent.

### Recruitment

Participants were recruited into the parent study in 2015–2016 by health department staff from patients who had received at least two weeks of treatment monitored by traditional in-person DOT and had at least 30 days remaining on their prescribed treatment regimen. Patients were invited to switch to VDOT for the remainder of their treatment course. Patients who refused to participate or did not meet eligibility criteria continued to have their treatment monitored through in-person DOT.

### Data collection

Data were collected during telephone interviews conducted with participants prior to initiating VDOT use (baseline) and again upon completion of VDOT use (follow-up; median duration = 5.4 months). Each interview took approximately 15 min to complete. Interviews were conducted by research staff, rather than healthcare providers to minimize potential socially desirable responses from participants. TB program staff were responsible for assigning phones to patients, teaching patients how to perform VDOT, watching patient videos to document adherence, and retrieving phones at the end of treatment.

The baseline questionnaire assessed patient characteristics that had the potential to differ by VDOT feasibility, acceptability and efficacy to improve treatment adherence. Smartphone ownership was defined as owning a touch screen phone that has access to the internet and other multimedia applications. To assess smartphone ownership at baseline, participants were first asked if they owned a cellular phone, and if “yes”, they were asked if it was a smartphone. Racial/ethnic groups consisting of less than 5% of the study population (i.e., Native Hawaiian/Pacific Islander, Alaskan Native, American Indian or mixed) were combined into an “other/mixed” category for this analysis.

Participant perceptions of their TB treatment using DOT and VDOT were assessed in follow-up interviews. Questions included level of satisfaction with VDOT, difficulty in using VDOT, preference for VDOT compared to DOT, and feelings towards the amount of interaction participants had with healthcare workers via VDOT and DOT. These questions allowed us to explore whether baseline smartphone ownership influenced participants’ responses to VDOT and whether these perceptions ultimately influenced the success of the intervention.

### Data analysis

A total of 151 participants were included in the parent study. Frequencies with percentages were computed for categorical variables and means with standard deviations were computed for continuous variables to describe the participants’ characteristics and VDOT perceptions. The outcome variable of interest was smartphone ownership (1 = not owning a smartphone, 0 = owning a smartphone). Univariate analyses were conducted using Chi-square or Fisher’s exact test for categorical variables and Wilcoxon rank sum test for continuous variables to identify factors associated with smartphone ownership. Simple logistic regression analysis was performed and unadjusted odds ratios with 95% confidence intervals were calculated. Baseline variables that were significantly associated with smartphone ownership in univariate analysis (*p* < 0.1) were included in a multivariable logistic regression analysis. The final model was built using manual backward elimination of nonsignificant variables retaining only variables with *p* < 0.05. Adjusted odds ratios and 95% confidence intervals for independent correlates of not owning a smartphone were calculated. Analyses were conducted using SPSS (IBM Corp. Released 2015. IBM SPSS Statistics for Mac, Version 23.0. Armonk, NY, USA).

## Results

Baseline participant characteristics are summarized in Table 1 . Of the 151 study participants, 54 (35.8%) were from San Diego, 49 (32.5%) were from San Francisco, and 48 (31.8%) were from New York City (Table 1). At baseline, 49 (30.4%) participants did not own a smartphone. The mean age of participants was 41 (range: 18–87) years and 41.1% were female. Most participants identified as Asian (45%) or Latino (29.8%), and 76.8% were born outside of the USA. Overall, participants had low socioeconomic status as 49.6% earned less than $10,000 United States Dollars (USD) annually, 57.6% had a high school education or less, and only 62.3% lived in their own home or apartment; however, no participant reported being homeless (data not shown) and 79.3% had health insurance at baseline. Cigarette smoking, alcohol use, marijuana use, intravenous drug use, and incarceration status were included as risk factors known to be associated with TB and were therefore included in the univariate analysis. Lifetime history of cigarette smoking and alcohol use in the past six months were common, but few participants reported smoking marijuana in the past six months or a lifetime history of injection drug use or incarceration.

**Table 1:** Univariate analysis of baseline characteristics by smartphone ownership among individuals receiving treatment for tuberculosis in San Diego, San Francisco, and New York City, 2015-2016^a^

		Owns a smartphone?			
Variable	Total	No	Yes	Odds Ratio (95% CI)^b^	*p*-value^c^
Study Site, *n* (%)					0.479
San Diego	54 (35.8%)	14 (30.4%)	40 (38.1%)	1.0	
San Francisco	49 (32.5%)	18 (39.1%)	31 (29.5%)	1.66 (0.72, 3.85)	
New York City	48 (31.8%)	14 (30.4%)	34 (32.4%)	1.18 (0.49, 2.81)	
Age (yrs), mean (SD)	40.7 (16.0)	52.3 (16.7)	35.6 (12.8)	1.08 (1.05, 1.11)	< 0.001
Sex					0.081
Female	62 (41.1%)	14 (30.4%)	48 (45.7%)	1.0	
Male	89 (58.9%)	32 (69.6%)	57 (54.3%)	1.93 (0.92, 4.02)	
Education, *n* (%)					< 0.001
> High school	62 (41.6%)	9 (19.6%)	53 (51.5%)	1.0	
High school or less	87 (58.4%)	37 (80.4%)	50 (48.5%)	4.35 (1.92, 10.0)	
Country of Birth, *n* (%)					0.780
United States	35 (23.2%)	11 (23.9%)	24 (22.9%)	1.0	
Mexico	19 (12.6%)	7 (15.2%)	12 (11.4%)	1.27 (0.39, 4.12)	
Other	97 (64.2%)	28 (60.9%)	69 (65.7%)	0.89 (0.38, 2.05)	
Race, *n* (%)					0.388
African American/Black	20 (13.2%)	6 (13.0%)	14 (13.3%)	1.0	
Caucasian/White	10 (6.6%)	3 (6.5%)	7 (6.7%)	1.0 (0.19, 5.24)	
Latino	45 (29.8%)	13 (28.3%)	32 (30.5%)	0.95 (0.30, 3.00)	
Asian	68 (45.0%)	19 (41.3%)	49 (46.7%)	0.91 (0.30, 2.70)	
Other^d^	8 (5.3%)	5 (10.9%)	3 (2.9%)	3.89 (0.70, 21.7	
Annual income, *n* (%)					0.021
> =$10,000	71 (50.4%)	16 (36.4%)	55 (56.7%)	1.0	
< $10,000	70 (49.6%)	28 (63.6%)	42 (43.3%)	2.29 (1.10, 4.77)	
Has health insurance, *n* (%)					0.276
No	31 (20.7%)	12 (26.1%)	19 (18.3%)	1.0	
Yes	119 (79.3%)	34 (73.9%)	85 (81.7%)	0.63 (0.28, 1.45)	
Where did participant live at time of study?, *n* (%)					0.365
Own home or apartment	94 (62.3%)	25 (54.3%)	69 (65.7%)	1.0	
Other person’s home or apartment	50 (33.1%)	19 (41.3%)	31 (29.5%)	1.69 (0.81, 3.52)	
Other^e^	7 (4.6%)	2 (4.3%)	5 (4.8%)	1.10 (0.20, 6.06)	
Hours worked per week, mean (SD)	23.3 (20.6)	16.8 (19.3)	25.9 (20.7)	0.98 (0.96, 0.996)	0.030
TB risk factors, *n* (%)					
Ever a cigarette smoker					0.357
No	84 (55.6%)	23 (50.0%)	61 (58.1%)	1.0	
Yes	67 (44.4%)	23 (50.0%)	44 (41.9%)	1.39 (0.69, 2.78)	
Smoked marijuana in past 6 months					0.379
No	139 (92.1%)	41 (89.1%)	98 (93.1%)	1.0	
Yes	12 (7.9%)	5 (10.9%)	7 (6.7%)	1.70 (0.51, 5.74)	
Ever injected drugs					0.546
No	149 (98.7%)	45 (97.8%)	104 (99.0%)	1.0	
Yes	2 (1.3%)	1 (2.2%)	1 (1.0%)	2.31 (0.14, 37.77)	
Ever incarcerated					0.659
No	140 (92.7%)	42 (91.3%)	98 (93.3%)	1.0	
Yes	11 (7.3%)	4 (8.7%)	7 (6.7%)	1.33 (0.37, 4.80)	
Used alcohol < 1 time per month in the past 6 months					0.458
No	108 (72.0%)	35 (76.1%)	73 (70.2%)	1.0	
Yes	42 (28.0%)	11 (23.9%)	31 (29.8%)	0.74 (0.33, 1.64)	

^a^Abbreviations: VDOT, video directly observed therapy; DOT, directly observed therapy; TB, tuberculosis.

^b^Odds ratios and confidence intervals were computed using simple logistic regression analysis.

^*c*^*P*-values are based on Chi-square tests, Fisher’s Exact test, or Wilcoxon test and examine overall significance of differences between smartphone ownership within the groups.

^d^Other includes: Native Hawaiian/Pacific Islander, Alaskan Native, American Indian, or Mixed.

^e^Other includes: Hotel or rooming house, shelter, welfare, boarding home

In univariate analysis (Table 1), income, education, hours worked/week, sex, and age were significantly associated with not owning a smartphone (*p* < 0.1). In multivariable analysis, not owning a smartphone was independently associated with older age, male sex, lower income, and lower educational attainment (Table 2). Hours worked/week and income were colinear causing the model containing both variables to be unstable; therefore, we selected income for the multivariable analysis because we believe it more accurately reflected the participant’s ability to purchase a smartphone. No other factors investigated had a statistically significant association with not owning a smartphone.

**Table 2:** Multivariable logistic regression analysis of baseline participant characteristics associated with not owning a smartphone among individuals receiving tuberculosis treatment in San Diego, San Francisco, and New York City, 2015–2016 (*n* = 123)^a^

Variable	Adjusted Odds Ratio (95% CI)	*P*-value
Age (yrs)	1.09 (1.05, 1.13)	0.000
Sex		
Female	1.00	
Male	2.86 (1.04, 7.86)	0.041
Education		
Above high school	1.00	
High school or below	4.48 (1.57, 12.80)	0.005
Annual Income		
≥ $10,000	1.00	
< $10,000	3.06 (1.19, 7.89)	0.020

^a^28 were excluded from the final multivariable analysis due to missing data on the baseline questionnaires for the variables included in the final model

Table 3 describes participants’ perceptions of DOT prior to the start of VDOT use and provides a comparison of VDOT to DOT at the end of VDOT use by smartphone ownership. Compared to smartphone owners, a higher proportion of non-smartphone owners reported that having a DOT worker visit and watch them take their medications made them feel cared for (13.0% vs. 1.0%; *p* = 0.001). Although few participants overall reported feeling that they that they did not have enough contact with their healthcare worker while using VDOT (4.8%), non-smartphone owners were more likely to report this than smartphone owners (10.5% vs. 2.3%, *p* = 0.034). Participants overall learned to use VDOT quickly (mean = 1.8 days of practice) and most participants preferred VDOT to DOT (84.0%) and found VDOT “very easy” to use (78.9%). Despite the differences in perception noted above, participants overall reported feeling “very satisfied” with their TB treatment monitoring via VDOT (69.6%), which did not differ by smartphone ownership status.

**Table 3:** Univariate analysis of treatment perceptions by smartphone ownership among individuals receiving treatment for tuberculosis in San Diego, San Francisco, and New York City between 2015-2016^a^

		Owns a Smartphone?		
Variable	Total	No	Yes	*p* value^b^
Having a DOT worker come to watch me take my TB medication makes me feel…, *n* (%) ^c^				
Like I am not trustworthy				0.474
No	143 (95.3%)	43 (93.5%)	100 (96.2%)	
Yes	7 (4.7%)	3 (6.5%)	4 (3.9%)	
Cared for				0.001
No	143 (95.3%)	40 (87.0%)	103 (99.0%)	
Yes	7 (4.7%)	6 (13.0%)	1 (1.0%)	
I don’t mind it				0.117
No	86 (57.3%)	22 (47.8%)	64 (61.5%)	
Yes	64 (42.7%)	24 (52.2%)	40 (38.5%)	
Patronized				0.65
No	145 (96.7%)	44 (95.7%)	101 (97.1%)	
Yes	5 (3.3%)	2 (4.4%)	3 (2.9%)	
Embarrassed				0.84
No	138 (92.0%)	42 (91.3%)	96 (92.3%)	
Yes	12 (8.0%)	4 (2.7%)	8 (5.3%)	
Practice days needed to learn to use VDOT application, mean (SD)	1.8 (3.0)	1.5 (1.4)	1.9 (3.5)	0.591
Preferred monitoring method, *n* (%)				0.111
VDOT	105 (84.0%)	34 (89.5%)	71 (81.6%)	
In-person DOT	1 (0.8%)	1 (2.6%)	0 (0.0%)	
No preference	19 (15.2%)	3 (7.9%)	16 (18.4%)	
Rate the amount of contact with healthcare worker during VDOT, *n* (%)				0.034
Too much	7 (5.6%)	0 (0.0%)	7 (8.0%)	
Just enough	112 (89.6)	34 (89.5%)	78 (89.7%)	
Not enough	6 (4.8%)	4 (10.5%)	2 (2.3%)	
Rate the ease of using VDOT, *n* (%)				0.691
Very easy	97 (78.9%)	29 (76.3%)	68 (80.0%)	
Somewhat easy	22 (17.9%)	7 (18.4%)	15 (17.6%)	
Somewhat difficult	4 (3.3%)	2 (5.3%)	2 (3.4%)	
Level of satisfaction with TB monitoring via VDOT, *n* (%)				0.679
Very satisfied	87 (69.6%)	25 (65.8%)	62 (71.3%)	
Somewhat satisfied	23 (18.4%)	9 (23.7%)	14 (16.1%)	
Neutral/indifferent	6 (4.8%)	1 (2.6%)	5 (5.7%)	
Somewhat or very dissatisfied	9 (7.2%)	3 (7.9%)	6 (6.9%)	

^a^Abbreviations: VDOT, video directly observed therapy; DOT, directly observed therapy; TB, tuberculosis

^b^*P*-values are based on Chi-square tests, Fisher’s Exact test, or Wilcoxon rank sum test and examine overall significance of differences between smartphone ownership within the groups.

^c^This variable was assessed at baseline prior to VDOT use. All other variables were assessed at follow-up after VDOT use

## Discussion

This study addressed the question of who would be denied access to mHealth interventions if they were only available to patients who possessed their own smartphone. We found that one-third of patients being treated for TB by health departments in three USA metropolitan cities did not own a smartphone prior to the study. Older age, male sex, lower income, and lower education level were associated with higher odds of not owning a smartphone. Importantly, however, we also found that smartphone ownership was unassociated with participants’ ability to learn or use the VDOT application.

Our finding that there is less ownership among older patients is consistent with other studies [13]. This is likely due to a combination of factors that includes the increased availability of smartphones in the last decade and the increased earning potential of younger participants that would allow them to purchase a smartphone [14]. We also found that men were more likely to not own a smartphone than women. While this was a surprising finding based on prior studies showing that women are less likely to own a smartphone than men, similar findings were observed in other studies [4–6, 15–17]. Although this finding was unexpected, it is reassuring to see that women, who historically had less access to technology, appear to have greater smartphone ownership in this cohort. Furthermore, we did not observe a significant difference in education level or income between male and female participants. Our results, while unexpected, are consistent with current trends showing that the gender gap in smartphone use is reversing in both high and low resource settings [16]. This could be due in part to the steadily decreasing wage-gender gap in the USA [18]. Women were found to make 80–90% per dollar compared to their male counterparts, up from 70% in the 1990s and early 2000s [18]. This rise is occurring in conjunction with the increasing labor market value of women and the increase in full-time workers [18]. Together these findings support the notion that more women would have a greater use for smartphones as well as the means to purchase them.

Not surprisingly, lower educational attainment and income were independently associated with non-smartphone ownership, as other studies have shown that smartphone ownership is more prominent among individuals with higher socioeconomic status [7, 19, 20]. When we analyzed education level by annual income (<$10,000 vs. ≥$10,000), we found that only 42.7% of participants with a high school education or below earned more than $10,000 a year compared to 60.3% of participants who completed more than a high school education. These results are consistent with other studies, including one that analyzed cellphone ownership among persons who inject drugs (PWID) in Tijuana, Mexico. The authors acknowledge that marginalized individuals were less likely to have the resources necessary to own and maintain a cellphone [21, 22]. While PWID cannot be directly compared to patients with TB, it is reasonable to expect that members of both groups have overlapping sociodemographic characteristics given that TB is often associated with poverty in high-burden settings [23].

Although most participants perceived their care to be good, we observed that participants who did not own a smartphone were more likely to report that in-person DOT made them feel “cared for” and that the amount of contact with healthcare workers during VDOT was “not enough”. There are multiple possible explanations for these findings. The observed associations could be because smartphone ownership is a proxy for having access to healthcare and patients with less access to healthcare (i.e., non-smartphone owners) might value the time spent with a healthcare provider during DOT visits more than patients who are accustomed to having access to their doctor (i.e., smartphone owners). Consistent with previous studies, those without smartphones were more likely to be older and belong to a low-income group, which could also contribute to greater social isolation [20, 24]. The preference for in-person DOT and more contact with treatment staff could therefore be valued for its effect on reducing isolation [20, 24]. Additionally, older or less educated patients might associate the in-person component of DOT with more attention from their provider and better care.

Despite these findings, differences in perceptions between the two groups did not affect participants’ ability to use VDOT. Notably, both groups predominantly found VDOT very easy to use and required less than two practice days to learn how to use the application. Moving forward, this should reassure designers of mHealth applications that baseline smartphone ownership is not necessary for successful use of the device with appropriate training. These results are important for designing future mHealth interventions in general and for understanding patient preferences in the methods used to monitor TB treatment adherence.

Some limitations should be considered when interpreting these findings. Since all study participants had used DOT before taking part in the study, it is possible that individuals who were willing to switch to VDOT were more likely to own a smartphone and more inclined to view VDOT as a positive intervention. However, only five patients in San Diego and three patients in San Francisco refused to participate, making selection bias and overestimation of smartphone ownership unlikely. It should also be noted that this study was limited to USA cities. Most of the global burden of TB is in other more resource-limited settings that may also have different levels of cellular phone and internet infrastructure as well as cultural and societal factors. Therefore, the results of this study might not be generalizable to other parts of the world. The study’s sample size potentially limited our ability to identify additional factors associated with smartphone ownership. Familiarity and comfort with using features on a smartphone could have varied among participants who reported owning a smartphone. For example, some participants might have only used it as a telephone, while others used it for calling, texting, emailing, Internet browsing, and engaging with other applications. The dependent variable (i.e., smartphone ownership) did not specify how participants used their smartphones. Finally, all study participants were TB patients receiving treatment through large urban public health departments, which might not generalize to other patient populations.

mHealth interventions requiring patients to use their own smartphone in the USA have the potential to disproportionately exclude patients who are male, older, and less educated. It is important to recognize that changing economic and societal norms have made access to smartphones more prevalent across the general population, thereby reducing the potential for the observed disparities. However, until these inequities are eliminated, there is still a concern that certain groups of patients could be systematically excluded if personal smartphone ownership is required. While recent studies in other parts of the world have suggested that mobile phone usage is prevalent enough to justify the use of mHealth interventions, it is not yet clear if or how outcomes will differ between VDOT and DOT, or whether providing smartphones to low-income patients will affect the impact of VDOT [8]. Similar studies conducted in rural areas and with other patient populations are also needed to determine whether these results are generalizable to the broader population. Healthcare providers proposing adoption of mHealth interventions should consider the availability of smartphones among the populations they serve and include contingencies to accommodate patients who lack smartphones to avoid creating or perpetuating health disparities.

## Conclusions

Although mHealth interventions for the treatment of TB and other diseases have gained popularity, some questions remain about their sustainability within resource-limited populations. In looking at barriers to smartphone ownership, we found that patients who were older, male, less educated, or had lower annual income were less likely to own smartphones and could be denied access to mHealth interventions if personal smartphone ownership is required. Though smartphone ownership has become more prevalent, it is important to bear in mind that certain groups might be excluded when designing similar mHealth interventions.

## Data Availability

The datasets used and/or analyzed during the current study are available from the corresponding author on reasonable request.

## Abbreviations

AOR: Adjusted Odds Ratio
CI: Confidence Interval
DOT: Directly Observed Therapy
mHealth: Mobile Health
PWID: Persons Who Inject Drugs
TB: Tuberculosis
USA: United States of America
USD: United States Dollars
WHO: World Health Organization
